# Temporal processing of past and future autobiographical events in patients with schizophrenia

**DOI:** 10.1038/s41598-019-50447-y

**Published:** 2019-09-25

**Authors:** Hédi Ben Malek, Arnaud D’Argembeau, Mélissa C. Allé, Nicolas Meyer, Jean-Marie Danion, Fabrice Berna

**Affiliations:** 1 Department of Psychology, Psychology and Neuroscience of Cognition Research Unit, University of Liège, Liège, Belgium; 2 Inserm U1114 - Cognitive Neuropsychology and Pathophysiology of Schizophrenia, Strasbourg, France; 3 University of Strasbourg, Strasbourg, France; 4 Center on Autobiographical Memory Research, Department of Psychology and Behavioural Sciences, Aarhus University, Aarhus, Denmark; 5 University Hospital of Strasbourg, Strasbourg, France

**Keywords:** Psychiatric disorders, Schizophrenia

## Abstract

People with schizophrenia experience difficulties in remembering their past and envisioning their future. However, while alterations of event representation are well documented, little is known about how personal events are located and ordered in time. Using a think-aloud procedure, we investigated which strategies are used to determine the times of past and future events in 30 patients with schizophrenia and 30 control participants. We found that the direct access to temporal information of important events was preserved in patients with schizophrenia. However, when events were not directly located in time, patients less frequently used a combination of strategies and partly relied on different strategies to reconstruct or infer the times of past and future events. In particular, they used temporal landmark events and contextual details (e.g., about places, persons, or weather conditions) less frequently than controls to locate events in time. Furthermore, patients made more errors when they were asked to determine the temporal order of the past and future events that had been previously dated. Together, these findings shed new light on the mechanisms involved in locating and ordering personal events in past and future times and their alteration in schizophrenia.

## Introduction

Patients with schizophrenia experience difficulties in remembering their past and imagining their future. Notably, there is substantial evidence that autobiographical memories lack contextual details and are less specific (i.e., referring to unique experiences happening at a specific place and time, and lasting no more than a day) in patients with schizophrenia than control participants^1^. Similarly, patients imagine future events that are less specific^2,3^ and less detailed^4^. Surprisingly, however, while the ability to consider times in the past and the future is an important component of ‘mental time travel’^5,6^, it remains unclear whether and how patients with schizophrenia present alterations of the sense of *when* events occurred or will occur. Given that the representation of time is crucial to the process of setting and pursuing personal goals^7^, investigating temporal location and order processes of personal events in schizophrenia may contribute to better understand why patients find it hard to set, plan and pursue personal goals. Therefore, the present study aimed to examine whether processes involved in the temporal location and order of personal past and future events are altered in schizophrenia.

Research has shown that three types of processes contribute to the ability to determine the times of past events: location-, order- and distance-based processes^8,9^. Location processes are used to place events at particular points in conventional (e.g., parts of days, months, years), natural (e.g., seasons), or personal (e.g., lifetime periods) time patterns; examples include recalling that an event happened on a weekend, during winter, or when one was in college. Order codes refer to before-after relations between events, which can be used to place events relative to each other. Finally, distance-based processes give rise to the impression that an event happened a long time ago or recently, which is in part determined by some properties of memories, such as their vividness.

Although all three processes can be used to date past events, people are especially adept at determining the temporal locations of past events^9^, and similar location-based processes are involved in envisioning the times of imagined future events^5,10^. According to reconstructive theories^11–13^, locations are often not intrinsic properties of memories but are reconstructed using multiple sources of information, for instance episodic information such as contextual details (i.e., persons, places, activities, or any other content) or specific events playing the role of temporal landmarks, and semantic information such as general knowledge of time patterns and events of one’s life (e.g., knowledge of autobiographical periods or extended events). However, in some cases, the dates of important events can be directly accessed (e.g., graduations, wedding, children’s birth), both for the past and the future^14–17^.

Little is known about temporal location processes in schizophrenia. Previous studies showed that the ability to consciously represent time information for personal events is reduced for past events^18^, but relatively spared for future events^19^. Regarding non-personal events, Venneri *et al*.^20^ showed that patients with schizophrenia make more dating errors and are less precise when they are asked to date historical events (for example, the fatal car accident of Princess Diana). These findings suggest that the dating of events might be (at least partly) altered in schizophrenia, although none of these studies examined the mechanisms involved in the ability to locate events in time. Thus, it remains unclear whether the mechanisms underlying the temporal location of personal events are impaired in schizophrenia.

Regarding temporal order processes, to our knowledge, no study specifically examined the ability to order personal events in schizophrenia. Nonetheless, it has been found that patients’ narratives of their life story^21,22^ or self-relevant life events^23^ are less temporally organized. Furthermore, a number of studies examined order performance for non-personal events. For example, some researchers used recency discrimination tasks in which participants were instructed to judge which of two items (e.g., words^24^, images^25^ or household objects^26^) was most recently memorized, and found that patients with schizophrenia exhibit poorer performance than control participants, suggesting an alteration of temporal order processes. Another study that used a picture-sequencing task yielded similar results^27^. While these findings indicate that temporal order processes are impacted for non-personal events (at least after a short delay), it remains unknown whether patients with schizophrenia are able to order events that are personal and more distant in time.

The first aim of the present study was to investigate temporal location processes in schizophrenia by examining the strategies that patients use to locate personal events in past and future times. Secondly, we sought to examine the capacity for patients to order personal past and future events in time. Based on the literature reviewed above, we expected that both temporal location and ordering processes would be altered in schizophrenia. More precisely, we expected that patients with schizophrenia would exhibit difficulty using episodic (but not semantic) information to date events and would make more errors when ordering past and future events in time, relatively to control participants.

## Method

### Participants

Thirty outpatients with schizophrenia (10 women) were recruited from the Department of Psychiatry of Strasbourg’s University Hospital, along with 30 control participants matched on gender, age and years of schooling. All the patients fulfilled the DSM-5 criteria^28^ for schizophrenia or schizo-affective disorder, and were clinically stabilized under antipsychotic medication. The mean dose equivalent to chlorpromazine was 320.6 mg/day (SD = 201.4). The mean duration of the illness was 13.78 years (SD = 7.18). The participants were all native French speakers. Exclusion criteria for both patients and controls were the following: severe somatic illness; history or current neurologic disorders (e.g., traumatic brain injury, epilepsy); psychiatric disorders (other than schizophrenia, for patients); current alcohol or substance abuse disorder; major depressive episode, defined for patients by a score higher than 6 on the Calgary Depression Scale for Schizophrenia^29^ (CDSS) and for controls as a score higher than 9 on the Beck Depression Inventory^30^ (BDI); and IQ score below 70 on the French validated short version of WAIS-III^31,32^ (Weschler Adult Intelligence Scale – third edition).

This study was approved by the Ethical Review Board South-East IV (reference 2016-A01463-48). All participants gave informed written consent to take part in the study. All methods were performed in accordance with the relevant guidelines and regulations.

### Materials and procedure

#### Clinical assessment

A full description of the clinical and neuropsychological measures is presented in Table 1. The severity of clinical symptoms of patients was assessed using the Positive And Negative Syndrome Scale^33^ (PANSS). Depression was checked with the CDSS for patients, and the BDI for controls. For both groups of participants, apathy was assessed using the Lille Apathy Rating Scale^34^ (LARS), and the level of anxiety was checked with the State-Trait Anxiety Inventory^35,36^ (STAI Y-A & Y-B).

**Table 1:** Means (and standard deviations) of clinical and neuropsychological measures for patients with schizophrenia (n = 30) and controls (n = 30).

	Control participants		Patients with schizophrenia		Statistics				
	n = 30		n = 30		θ		*CI* 95%		*Pr*(θ > 0)
	*M*	*SD*	*M*	*SD*	*M*	*SD*	2.5%	97.5%	
**Clinical measures**									
Age	37.8	10.1	37.3	9.6	0.92	2.5	−4.0	5.9	0.641
Gender (number of women)	10	33.3%	10	33.3%					
Years of schooling	13.2	2.3	12.1	2.2	−1.1	0.6	−2.2	0.1	0.038
LARS (apathy)	−25.6	6.4	−18.1	5.9	7.0	1.6	3.8	10.2	>0.999
STAI Y-A (state anxiety)	42.7	6.7	49.9	9.6	8.4	2.1	4.2	12.6	>0.999
STAI-Y-B (trait anxiety)	41.4	8.2	48.2	9.6	7.5	2.3	3.0	12.0	>0.999
Depression (BDI)	3.03	3.43							
Depression (CDSS)			2.0	2.4					
PANSS total			53.6	16.2					
PANSS positive			12.2	3.8					
PANSS negative			17.5	8.2					
**Psychometric measures**									
fNART (pre-morbid IQ)	111.0	6.1	104.7	10.9	−2.6	2.4	−7.2	2.4	0.142
WAIS-III (current IQ)	101.1	13.0	86.5	13.5	−6.3	3.9	−13.7	1.97	0.063
**Neuropsychological measures**									
WAIS IV cancellation	10.4	2.6	7.3	2.5	−3.1	0.7	−4.4	−1.7	<0.001
WAIS IV direct digit span	9.8	2.1	8.8	2.7	−1.0	0.6	−2.3	0.2	0.053
WAIS IV reverse digit span	10.0	2.8	8.3	2.8	−1.6	0.7	−3.1	−0.2	0.015
Fluency phonological	17.7	6.0	18.4	6.7	0.9	1.7	−2.5	4.4	0.709
Fluency semantic	21.9	5.5	18.2	5.4	−3.0	1.4	−5.8	−0.1	0.020
TMT A – B (time, in seconds)	34.1	17.8	67.5	58.2	23.6	7.3	9.1	37.7	>0.999
TMT A – B (number of errors)	0.1	0.7	0.9	1.6	0.8	0.3	0.1	1.4	0.990

*Note*: Results are presented as θ with a 95% Credible Interval (CI), with the probability of the θ being above 0: *Pr*(θ > 0).

LARS: Lille Apathy Rating Scale, STAI: State-Trait Anxiety Inventory, BDI: Beck Depression Inventory, CDSS: Calgary Depression Scale for Schizophrenia, PANSS: Positive And Negative Syndrome Scale, fNART: French National Adult Reading Test, WAIS: Weschler Adult Intelligence Scale, TMT; Trail Making Test.

#### Neuropsychological assessment

Pre-morbid and current IQ were assessed using the French validated version of the National Adult Reading Test^37^ (f-NART) and the WAIS-III short version, respectively. This short version included subtests of vocabulary, matrix reasoning, and arithmetic. Executive functioning was evaluated using the Trail-Making Test^38^ (TMT A & B), and phonologic and semantic fluency^39^. Processing speed was tested using the cancellation subtest of WAIS-IV^40^. Short-term memory and working memory were evaluated by the direct and reverse digit span subtest of WAIS-IV, respectively. Verbal fluency was assessed, and played a two-fold role of a measure of executive functioning^39^ and an interfering activity between temporal location and temporal order tasks (see below). The participants had 2 minutes to give as many words as they could starting with the letter “r” (phonological fluency), and 2 minutes to give as many fruit names as possible (semantic fluency).

#### Temporal location task

Participants were asked to think aloud while they attempted to locate a series of past and future events in time. The experimental task was inspired by previous work on past^14,41–43^ and future event dating^10,14^. The temporal location task involved three phases. First, participants had to retrieve 10 past events and to imagine 10 events that are likely to happen in the future, in response to cue-words (event-generation phase). Twenty cue words referring to common places and objects (e.g., book, house, coffee-shop, dog) were divided into 2 lists of 10 cues that were matched for frequency of use and imageability^44^. The allocation of the two lists to the past and future conditions and the order of presentation of the two conditions were counterbalanced across participants. For each cue word, participants were instructed to remember or imagine a specific personal event (i.e., a unique event happening at a specific place and time and lasting no longer than a day)^45^. There was no time limit to remember or imagine an event, but participants rarely exceeded two minutes to generate an event. A brief description of each generated event was written down by the experimenter.

Immediately after the event-generation phase, the descriptions of past and future events that had been evoked were presented one at a time and, for each event, participants were asked to describe everything that came to their minds (i.e., to think aloud^46^) while they attempted to determine as precisely as possible when the event occurred (past condition) or will likely occur (future condition) (event-dating phase). To avoid influencing temporal location processes, the instructions did not specify which type of temporal information should be expected (e.g., days, months, years). We considered that an event was located in time if the participant could provide at least the year during which the event happened (past condition) or would happen (future condition); note, however, that the majority of the temporally located events received a more precise temporal location. All verbal protocols collected during the think-aloud procedure were audio-recorded. For each trial, participants were also asked to rate their degree of certainty in the reported temporal location on a 7-point Likert scale (from 1 = extremely weak, to 7 = extremely strong).

After having located all events in time, participants were asked to rate each event on several 7-point Likert scales: the clarity of event representation (from 1 = not at all clear, to 7 = extremely clear), emotional valence (from -3 = very negative, to + 3 = very positive, with 0 = neutral), importance for personal goals (from 1 = not important at all, to 7 = very important), sense of mental time travel (from 1 = not at all, to 7 = totally), subjective temporal distance (from 1 = very close, to 7 = very distant), previous thought about the event (from 1 = never, to 7 = very often), previous thought about when the event occurred or would occur (from 1 = never, to 7 = very often), likelihood of future events (from 1 = not likely to happen, to 7 = very likely to happen).

#### Temporal order task

In the temporal order task, participants were instructed to order chronologically the past and future events that were previously produced. To do so, they had to place each event on an arrow of time (which only indicated the past, present and future), drawn on a blank sheet of paper, by writing keywords referring to the event. The list of past and future events was first read aloud by the experimenter and was then given to participants. To score temporal order performance, the temporal locations that were previously provided by participants were taken as reference. Thus, we compared the expected order of events (according to the dates determined in the temporal location task) to the order given by participants and we computed percentages of order errors for past and future events.

### Scoring

All the audio-recorded verbal protocols obtained while participants attempted to locate events in time were transcribed for scoring. When the temporal location of an event was immediately produced (i.e., without using any strategy), this was scored as direct event dating. When the temporal location was not directly produced, we scored the strategies used by the participants during the event-dating phase. To characterize these dating strategies, we used a scoring grid previously designed to classify the dating strategies of past and future events^14^. Five categories of strategies were considered: (1) lifetime periods/extended events, (2) specific events (landmarks), (3) conventional time patterns, (4) factual information, and (5) contextual details (for the definition of each category and examples of corresponding verbal reports, see Table 2). These five categories were not mutually exclusive (i.e., the dating protocol obtained for a particular event could include more than one type of strategy) and each trial was scored for the presence or absence of each category. Events that were not located in time were scored as uncategorized.

**Table 2:** Definition and examples of categories of temporal location strategies for past and future events.

Location strategy	Definition	Examples
Lifetime periods/extended events	Use of knowledge about lifetime periods or extended events for attempting to locate the event in time	It was during my Master’s degree (past event); It will happen during my internship (future event)
Specific events (landmarks)	Use of another specific event for which the precise temporal location is known (i.e., temporal landmark)	I met John a few days after my 25^th^ birthday (past event); It would be just before my thesis defence which is scheduled on the 1^st^ of November 2016 (future event)
Contextual details	Use of event details (such as locations, activities, persons, or the weather) to infer its temporal location	I was with François that day, so it certainly happened one month ago (past event); It has to be snowy, so it will likely happen in December (future event)
Conventional time patterns	Reasoning using calendar time (weeks, months, years) or natural time patterns (e.g., seasons)	It was a Monday, during this year, on October or November but I would say on October (past event); It will happen during the 1^st^ or the 2^nd^ week of July, more likely the first days of July (future event)
Factual information	Use of general knowledge (about self, others, or the world) to infer the temporal location of the event	At that time, my brother was still a baby, he is 6 years younger than me so it was on July 2005 (past event); To avoid mass tourism, I will go there during the 1^st^ week of September (future event)

All transcriptions were scored by the first author (HBM) and the reliability of our coding scheme was assessed by asking the third author (MA, who was trained for scoring and blind to diagnosis and hypothesis) to score a random selection of 15% of the verbal protocols. Percentages of raw agreements showed substantial inter-rater reliability for direct dating (95.4%) and for the five strategies of interest: 96.5% for lifetime periods/extended events, 95.9% for specific events, 95.9% for conventional time patterns, 93.6% for factual information, and 90.7% for contextual details. The Cohen’s kappa coefficients were not computed because the marginal distributions were not uniform^47^.

### Statistical analyses

Statistical analyses were performed using Bayesian methods. We used the R software (version 3.4.3) and the rjags package^48^. Univariate linear regressions were used to compute the between-group differences for clinical, psychometric and cognitive measures. Concerning the temporal location task, multilevel (with events as level 1 units, and participants as level 2 units) logistic regressions were used to analyze the influence of two predictors on the use of dating strategies: group (patients vs. controls), and time orientation (past vs. future). A multilevel Beta regression was computed to analyze the effect of the group and time orientation on order errors in the temporal order task. To compare the characteristics of directly dated events and events for which temporal information was reconstructed, we computed separate multilevel Beta regression analyses (for each characteristic) including two predictors, the group (patients vs. controls) and the mode of location (direct vs. reconstruction). Non-informative priors were used to analyze group effects. We used informative priors for time orientation and mode of location factors based on our previous findings^14^ (see Supplementary material), and then tested the robustness of results by means of sensitivity analyses using both non-informative and pessimistic priors. Correlation analyses were performed to investigate associations between temporal location strategies, order errors, the level of clinical symptoms and cognitive functioning.

To interpret the results, we considered both large *Pr*(OR > 1) values (i.e., >0.95) and small values of *Pr*(OR > 1) (i.e., <0.05) as reflecting meaningful effects of the factor under consideration.

## Results

While the two groups were matched for age, patients had about 1 year of schooling less than controls (see Table 1). Concerning clinical measures, patients reported higher levels of apathy and anxiety than controls. Concerning the cognitive measures, the pre-morbid IQ did not differ between the two groups, but the current IQ was lower in patients. Overall, patients with schizophrenia had worse executive functioning (except for phonological fluency), working memory, processing speed, and logical reasoning capacities than control participants.

### Frequency of unlocated events

In total, 277 past and 277 future events were included in the analyses for patients, and 299 past and 284 future events for controls; 11 additional events (5 past and 4 future events for patients, 2 future events for controls) were excluded because they did not meet the specificity criterion (i.e., a unique event happening at a specific place and time, and lasting no more than a day) and participants failed to produce an event on 52 of trials. Patients with schizophrenia were not able to date 1.5% (vs. 0.3% for controls) of past events and 12.3% (vs. 5% for controls) of future events. The difference between groups was not meaningful (OR = 16.93, CI95%:0.54-108.19, *Pr*(OR > 1) = 0.92). In both groups, the frequency of unlocated events was higher for the future than the past (OR = 57.72, CI95%: 3.55–346.68, *Pr*(OR > 1) >0.99). There was no interaction between group and time orientation (OR = 1.25, CI95%:0.03–6.00, *Pr*(OR > 1) = 0.37).

### Direct retrieval vs. reconstruction of temporal location

**Figure 1:**
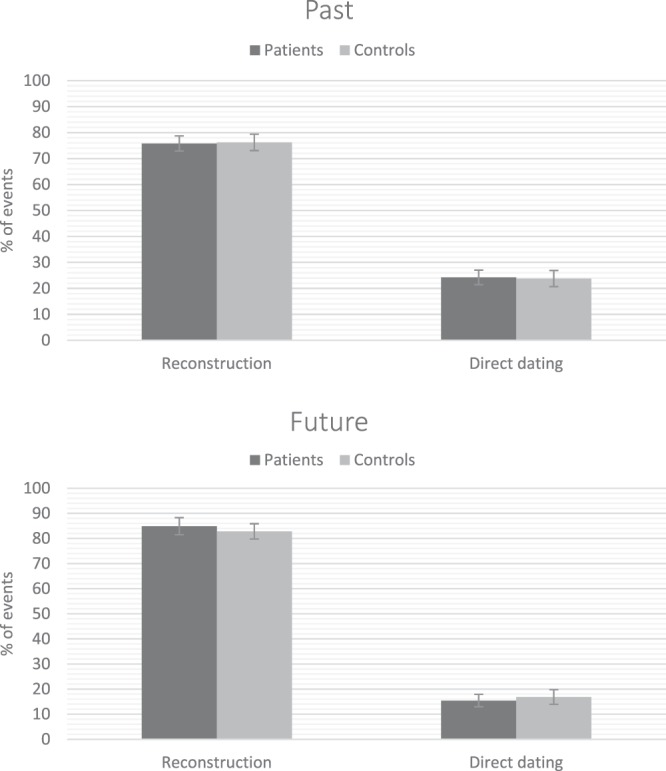
Mean percentages (and standard errors of the mean) of past and future events that were located in time using reconstructive strategies or direct dating for patients with schizophrenia (n = 30) and controls (n = 30).

As expected, the majority of events were located in time using reconstructive or inferential strategies (see Fig. 1). However, contrary to our expectation, the percentage of directly dated events did not differ between the two groups (OR = 1.06, CI95%:0.65–1.65, *Pr*(OR > 1) = 0.55). There was a meaningful effect of time orientation (OR = 0.63, CI95%:0.40–0.95, *Pr*(OR > 1) = 0.03), showing that direct access to dates was more frequent for past than future events. There was no interaction between group and time orientation (OR = 0.78, CI95%:0.40–1.39, *Pr*(OR > 1) = 0.18).

We also investigated whether the certainty with which participants located events in time differed as a function of the group and of their mode of location (direct vs. reconstruction). We found a meaningful effect of the group (OR = 0.63, CI95%:0.47–0.83, *Pr*(OR > 1) < 0.001), showing that the degree of certainty of temporal location was lower in patients (*M* = 5.14, *SD* = 1.42) than controls (*M* = 5.67, *SD* = 1.40). The effect of mode of location was meaningful (OR = 2.12, CI95%:1.69–2.64, *Pr*(OR > 1) >0.99), showing that directly dated events were judged as more certain (*M* = 6.33, *SD* = 0.98) than events located in time using reconstructive or inferential strategies (*M* = 5.18, *SD* = 1.44). No interaction was found between group and mode of location (OR = 1.18, CI95%:0.84–1.62, *Pr*(OR > 1) = 0.83).

### Frequency of reconstructive strategies

**Figure 2:**
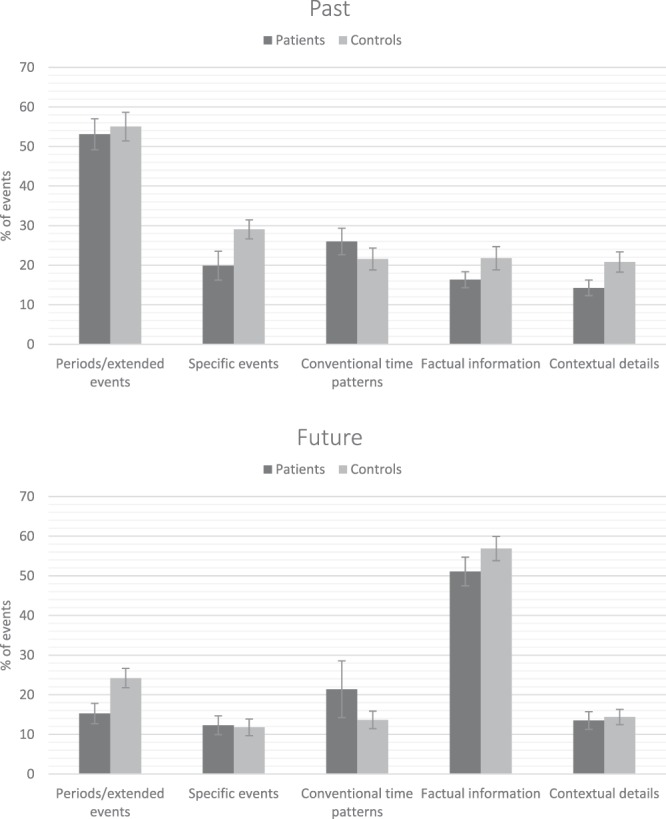
Mean percentages (and standard errors of the mean) of temporal location strategies for past and future events, for patients with schizophrenia (n = 30) and controls (n = 30).

To determine whether patients with schizophrenia relied on different reconstructive strategies to locate past and future events in time, we compared the percentage of use of strategies between groups and time orientations. As can be seen in Fig. 2, while participants in both groups used several strategies to locate past and future events in time, they most frequently used lifetime periods/extended events to date past events and factual information to date future events. Statistical analyses showed that patients used contextual details (OR = 0.67, CI95%:0.39–1.07, *Pr*(OR > 1) = 0.04) and specific landmark events (OR = 0.66, CI95%:0.40–1.02, *Pr*(OR > 1) = 0.03) less frequently than controls, and also tended to use factual information less frequently (OR = 0.71, CI95%:0.42–1.13, *Pr*(OR > 1) = 0.07); no meaningful between-group difference was found for lifetime periods/extended events and conventional time patterns (all *Pr*s(OR > 1) >0.30). Concerning time orientation, the use of lifetime periods/extended events (OR = 0.23, CI95%:0.15–0.34, *Pr*(OR > 1) < 0.001), specific events (OR = 0.39, CI95%:0.23–0.61, *Pr*(OR > 1) < 0.001), conventional time patterns (OR = 0.56, CI95%:0.33–0.90, *Pr*(OR > 1) = 0.008), and contextual details (OR = 0.65, CI95%:0.38–1.02, *Pr*(OR > 1) = 0.03) was less frequent for future events than for past events. On the other hand, the use of factual information was more frequent for future than past events (OR = 4.88, CI95%:3.19–7.22, *Pr*(OR > 1) =  >0.999). There was no interaction between group and time orientation for the frequency of use of any of the reconstructive strategies (all *Prs*(OR > 1) between 0.13 and 0.91).

We also examined whether the use of multiple (i.e., two or more) strategies differed between groups and time orientations. Results showed that the use of multiple strategies was less frequent for patients than controls (OR = 0.37, CI95%:0.17–0.70, *Pr*(OR > 1) < 0.001), and less frequent for future than past events (OR = 0.27, CI95%:0.17–0.42, *Pr*(OR > 1) < 0.001). On average, patients with schizophrenia used multiple strategies for 26% (*SD* = 25) of past events and 13% (*SD* = 17) of future events, whereas controls used multiple strategies for 44% (*SD* = 22) of past events and 20% (*SD* = 21) of future events. No interaction between group and time orientation was observed (OR = 1.43, CI95%:0.65–2.71, *Pr*(OR > 1) = 0.78). The most frequently used combination of strategies was lifetime periods/extended periods and factual information for patients (used for 37% of events, *SD* = 36; vs. 18% of events for controls, *SD* = 20) and lifetime periods/extended events and contextual details for controls (for 32% of events, *SD* = 34; vs. 10% of events for patients, *SD* = 14). For percentages of use of each combination of strategies for patients and controls, see Supplementary material.

### Event characteristics

The mean ratings of event characteristics are presented in Table 3, as a function of group and mode of location (directly located vs. reconstructed). Statistical analyses showed that patients provided lower ratings than controls for affective valence (OR = 0.73, CI95%:0.59–0.90, *Pr*(OR > 1) = 0.002), mental time travel (OR = 0.75, CI95%:0.50–1.07, *Pr*(OR > 1) = 0.05), and likelihood of future events (OR = 0.64, CI95%:0.46–0.88, *Pr*(OR > 1) = 0.003). There was no meaningful effect of group for subjective vividness, importance for personal goals, event and time rehearsal, subjective temporal distance and temporal location (all *Prs* (OR > 1) >0.11).

**Table 3:** Mean ratings (and standard deviations) of event characteristics for directly dated and temporally reconstructed events in patients with schizophrenia (n = 30) and controls (n = 30).

	Control participants n = 30				Patients with schizophrenia n = 30			
	Direct		Reconstruction		Direct		Reconstruction	
	*M*	*SD*	*M*	*SD*	*M*	*SD*	*M*	*SD*
Subjective vividness	5.9	1.2	4.8	1.1	4.9	1.3	4.5	0.9
Affective valence	1.6	1.3	1.5	0.6	1.3	1.3	1.2	0.7
Importance for personal goals	5.2	1.5	4.9	1.0	4.5	1.8	4.5	0.9
Mental time travel	5.5	1.2	4.6	1.1	4.5	1.1	4.2	0.9
Event rehearsal	3.7	1.0	3.6	0.8	3.4	1.3	3.3	0.8
Time rehearsal	3.6	1.1	3.3	0.9	3.3	1.5	3.1	0.8
Subjective temporal distance	2.7	1.0	3.9	0.7	3.2	1.2	3.8	0.7
Temporal location (months)	58.3	64.8	96.6	56.2	67.4	84.3	89.4	64.5
Likelihood (for future events)	6.1	0.9	5.3	0.8	5.7	1.2	4.9	0.8

Note. All dimensions were assessed on a Likert scale ranging from 1 to 7, except affective valence, which was assessed on a Likert scale ranging from −3 to 3, and temporal location from the present (which was assessed in months).

Concerning the mode of location, we found that directly located events received higher ratings on subjective vividness (OR = 2.03, CI95%:1.60–2.56, *Pr*(OR > 1) >0.99), importance for personal goals (OR = 1.33, CI95%:1.04–1.69, *Pr*(OR > 1) >0.99), mental time travel (OR = 1.89, CI95%:1.50–2.36, *Pr*(OR > 1) >0.99), likelihood of future events (OR = 2.07, CI95%:1.42–2.95, *Pr*(OR > 1) >0.99), and lower ratings on subjective temporal distance (OR = 0.44, CI95%:0.34–0.56, *Pr*(OR > 1) = < 0.001), compared to events that were located using reconstructive or inferential strategies. There was no difference between the two types of events for affective valence, event and time rehearsal, and temporal location (all *Prs* (OR > 1) between 0.07 and 0.91).

Finally, there were meaningful interactions between group and mode of location for subjective vividness (OR = 0.62, CI95%:0.44–0.87, *Pr*(OR > 1) = 0.002) and subjective temporal distance (OR = 1.42, CI95%:0.99–1.98, *Pr*(OR > 1) = 0.97), showing that directly dated events were judged more vivid and less temporally distant than events whose dates were reconstructed, in controls but not in patients. There was also an interaction for mental time travel (OR = 0.70, CI95%:0.41–0.96, *Pr*(OR > 1) = 0.01), showing that mental time travel was lower in patients than controls for directly dated events, but not for events whose dates were reconstructed.

### Temporal order

The percentage of events that were incorrectly ordered in time was compared between groups and time orientations. Results showed that order errors were more frequent in patients than controls (OR = 1.80, CI95%:1.03–2.96, *Pr*(OR > 1) = 0.98), but did not differ between past and future events (OR = 1.34, CI95%:0.80–2.12, *Pr*(OR > 1) = 0.86). No relevant interaction was found (OR = 0.75, CI95%:0.35–1.14, *Pr*(OR > 1) = 0.16). On average, patients with schizophrenia made order errors for 17% (*SD* = 16) of past events (vs. 7%, *SD* = 9, for controls) and 16% (*SD* = 16) of future events (vs. 10%, *SD* = 8, for controls).

Next, we computed the correlations between the percentage of use of temporal location strategies and percentage of events that were incorrectly ordered in time. We found that a more frequent use of lifetime periods/extended events was associated with an increase of order errors in patients (ρ = 0.42, CI95%:0.08–0.69, Pr(ρ > 0) = 0.99). The four other temporal locations strategies were not associated with order errors, and no relevant correlations were observed in controls (all Prs (ρ > 0) between 0.11 and 0.90).

### Relations with clinical symptoms and cognitive measures

We computed correlations between the percentage of use of each strategy/multiple strategies and the PANSS scores. There was no association between the PANSS total score and the use of any temporal location strategies (all *Prs* (ρ > 0) between 0.09 and 0.91). However, we found that a higher level of clinical symptoms was associated with a reduced use of multiple strategies (ρ = −0.31, CI95%:(−0.63)−(0.06), *Pr*(ρ > 0) = 0.04). This association was mainly due to the level of negative symptoms (ρ = −0.31, CI95%:(−0.62)−(0.07), *Pr*(ρ > 0) = 0.05), rather than positive symptoms (ρ = −0.16, CI95%:(−0.53)−(0.07), *Pr*(ρ > 0) = 0.16).

To assess the influence of intellectual and cognitive functioning in the frequency of use of reconstructive strategies (temporal location task), we computed additional statistical analyses in which current IQ, working memory (inverse digit span score), logical reasoning (matrix score), and years of schooling were entered as linear predictors in the statistical models. The results described above remained unchanged when IQ was taken into account, notably the group effect for specific landmark events (OR = 0.66, CI95%:0.38–1.08, *Pr*(OR > 1) = 0.04) and contextual details (OR = 0.61, CI95%:0.32–1.04, *Pr*(OR > 1) = 0.03). However, controlling for working memory lowered the group effect for landmark events (OR = 0.75, CI95%:0.45–1.18, *Pr*(OR > 1) = 0.10) and contextual details (OR = 0.75, CI95%:0.43–1.21, *Pr*(OR > 1) = 0.11). Controlling for logical reasoning also reduced the group effect for contextual details (OR = 0.98, CI95%:0.53–1.64, *Pr*(OR > 1) = 0.40), but not for landmark events (OR = 0.67, CI95%:0.38–1.09, *Pr*(OR > 1) = 0.05). Finally, controlling for years of schooling did not influence the group effect for landmark events (OR = 0.67, CI95%:0.40–1.04, *Pr*(OR > 1) = 0.03) but slightly lowered the group effect for contextual details (OR = 0.71, CI95%:0.41–1.14, *Pr*(OR > 1) = 0.07).

In a similar manner, we conducted additional statistical analyses to assess the influence of intellectual and cognitive functioning on the percentage of events that were incorrectly ordered in time (temporal order task). Group effects were reduced when current IQ (OR = 1.34, CI95%:0.70–2.34, *Pr*(OR > 1) = 0.79) and logical reasoning (OR = 1.42, CI95%:0.78–2.41, *Pr*(OR > 1) = 0.86), and to a lesser extent working memory (OR = 1.56, CI95%:0.88–2.57, *Pr*(OR > 1) = 0.93) and years of schooling (OR = 1.58, CI95%:0.89–2.63, *Pr*(OR > 1) = 0.94), were entered in the analysis.

### Sensitivity analyses

We tested the robustness of the statistical analyses using non-informative and pessimistic priors (i.e. informative priors used in the opposite direction of the expected effect) for time orientation and mode of location, and our conclusions remained globally unchanged (for description of the few changes, see Supplementary material).

## Discussion

The aim of the present study was to investigate temporal location and order processes for past and future events in schizophrenia. Our results showed that patients directly accessed to the temporal location of important events as frequently as control participants. However, when events were not directly dated, patients with schizophrenia less frequently relied on a combination of strategies and used contextual details and temporal landmark events less frequently than control participants to reconstruct or infer the dates of personal events. Moreover, patients with schizophrenia were less certain about the given dates and made more errors when they were later asked to temporally order events in time. Taken together, these results shed new light on the temporal location and order processes that are altered in schizophrenia.

In line with previous studies^10,14–17^, we found that the majority of past and future events were located in time using reconstructive processes, and that only a minority of events were directly located in time. This preponderance of reconstructive strategies was observed in both groups of participants. However, we found that patients with schizophrenia relied on a combination of strategies less frequently than controls to reconstruct or infer the dates of past and future events. A possible explanation for the reduced use of a combination of strategies may be that patients have difficulties to use some of these strategies. Indeed, we found that the proportion of use of contextual details and temporal landmark events was lower in patients than in controls, and that patients mainly relied on the combination of semantic strategies (i.e., lifetime periods, factual information) rather than episodic strategies (i.e., specific landmark events, contextual details) as controls did. Contextual details are an important source of information that is frequently used by healthy people to estimate the times of past events^14^. Temporal landmarks are meaningful and vivid events (such as one’s graduation, children’s birth, and so on) which date is known; such events contribute to structure past and future subjective times^12^ and to determine the temporal location of other events^9,13^. The less frequent use of these strategies and their combination to locate past and future events in time may be explained by patients’ reduced ability to access episodic information in long-term memory^1,49^. The lower feeling of mental time travel observed in patients corroborates this deficient access to episodic details, which could not be used to reconstruct or infer the times of past and future events.

Correlation analyses further revealed that patients’ reduced capacity to combine strategies to date events was more marked in patients with higher levels of symptoms, in particular negative symptoms. This result aligns with previous studies showing an association between the severity of negative symptoms and the capacity to access episodic memory details^4,23^. Additional analyses also suggested that impairments of working memory (and to a lesser extent of logical reasoning) may partly contribute to explain the deficient access to episodic information to reconstruct or infer the times of personal events in schizophrenia. An alternative hypothesis for the lack of use of specific landmark events could be that patient with schizophrenia experienced fewer events that would be considered as landmarks (relative to control participants), due to a reduced social, personal or professional activity. However, this alternative view would not explain why patients with schizophrenia had a reduced access to contextual details to reconstruct or infer the dates of personal events. Therefore, we think that a deficient access to episodic information in long-term memory—in part due to working memory deficits—better explains our results.

Besides these differences in the use of contextual details and landmark events, patients relied on semantic and general knowledge (i.e., lifetime periods/extended events, factual information, conventional time patterns) to the same extent as controls to reconstruct or infer the times of past and future events. Previous studies have shown that knowledge about lifetime periods is frequently used to date personal events^10,14^. Indeed, lifetime periods contextualize specific events in one’s personal life story^50^ and contain temporal knowledge that can be used to retrieve or envision the dates of past or future events^14,51^. Holm *et al*.^52^ showed that patients with schizophrenia are able to narrate and to date chapters of their life story. This preserved access to autobiographical periods indicates that some basic, easily accessible, and coarse temporal organisation of past and future thought may be preserved in schizophrenia.

Our results also showed that patients with schizophrenia were less certain than controls about the temporal locations of past and future events. This aligns with previous results showing that patients’ ability to clearly remember when personal events happened is affected^18^. However, it is worth mentioning that the certainty ratings of patients were still relatively high (*M* = 5.14 on a 7-point scale, compared to *M* = 5.67 in controls), suggesting that patients did not date events at random. Interestingly, we found that patients with schizophrenia made more errors than controls when they were later asked to temporally order the past and future events that had been previously dated. A possible explanation could be that the temporal locations provided by patients were not reliable, which lead to an increase of errors when they had to temporally order the same events. Another explanation would be that the provided dates were reliable, but that temporal order processes are altered in schizophrenia. A limitation of the present study is that we cannot distinguish between these two explanations because we did not collect independent information that would allow us to check whether the provided dates were accurate. Interestingly, however, we found that the use of lifetime periods/extended events to locate events in time was associated with an increase of order errors in patients, which suggests that the dates inferred from the knowledge of lifetime periods may not be precise enough to correctly order the events in time. This is in line with previous findings showing that the use of lifetime periods was associated with a reduced accuracy of the dating of past events, compared to the use of specific landmark events^16,17^.

Additional analyses further suggested that temporal order processes may be altered in schizophrenia due to more basic cognitive dysfunctions. In fact, our results showed that intellectual efficiency, logical reasoning capacities, and to a lesser extent working memory capacities accounted for the difficulty for patients to order events in time. Taken together, our findings suggest that the difficulty of patients with schizophrenia to order personal past and future events in time may relate to their cognitive dysfunction, as well as their propensity to use coarser temporal location processes, which might contribute to blur their representation of past and future times.

Interestingly, however, the present findings showed that patients with schizophrenia were able to directly locate past or future events in time as frequently as controls, and the proportion of directly dated events (between 15 and 25%) in both groups was similar to that reported in previous studies^10,14–17^. It is worth mentioning that the minority of events that are directly located usually correspond to personally important and temporally close events^8,9,13,14^, and the direct access to temporal information for these events may be critical for successful goal pursuit^10^. In line with this view, we found that both patients and controls rated those events as more important for personal goals (and more likely to happen for future events) than events whose dates were reconstructed, suggesting that knowledge about personal goals may facilitate access to temporal information^10^ and supporting the view that time and goal processes are intimately linked^7^. Nonetheless, the subjective vividness and feeling of mental time travel were higher for directly located events compared to reconstructed events in controls but not in patients. According to the model of Self-Memory System^53^, personal goals facilitate access to episodic information both when remembering the past and when imagining the future^54^. The lower vividness of directly dated events in patients may reflect a reduced influence of goals on episodic access and/or a weakening of central control processes guiding the access to autobiographical memory in schizophrenia, a hypothesis discussed elsewhere^55^.

Some limitations of this study should be acknowledged. First, we did not collect independent information that would allow us to check whether the dates provided for past and future events were accurate. A first reason is that it would be difficult to check the accuracy of dates for remote memories, and it would take a potentially long time to check whether imagined future events actually happen. A second reason is that our aim was to unravel the mechanisms engaged in the temporal location of autobiographical events, and not to determine whether these mechanisms influence the precision of dates. Another issue that should be acknowledged concerns the use of the think-aloud procedure^46^; our experimental task was inspired by previous work on past^41–43^ and future^10,14^ event dating that used a similar think-aloud method. One could argue that thinking aloud might alter temporal location processes and that verbalization might not accurately reflect the underlying location processes because participants might not report some thoughts or, conversely, might report mental events that did not occur^56^. Converging evidence using other measures of temporal location processes (e.g., response times, self-rating of the use of strategies, diary protocol) are thus needed to confirm the present findings.

To conclude, the present study showed that patients with schizophrenia exhibit some alterations of temporal location processes. They less frequently use combinations of strategies and strategies based on episodic information to reconstruct or infer the times of past and future autobiographical events, in comparison to controls. They also exhibit greater difficulty to order personal events in time. These findings suggest that the temporal component of mental time travel is blurred in schizophrenia and point to possible therapeutic implications. For instance, training patients to better access episodic information (e.g., by providing relevant cues or using visual imagery) and helping them to better specify and organize future times may contribute to improve personal goal pursuit.

## Supplementary information

Supplementary material

## Data Availability

The datasets of the study are available from the corresponding author on reasonable request.
