# Supplementary material for: Temporal processing of past and future autobiographical events in patients with schizophrenia
Source: Sci Rep. 2019 Sep 25;9:13858. doi: 10.1038/s41598-019-50447-y (PMC6761096; doi:10.1038/s41598-019-50447-y)
Supplement: Supplementary file 1 — Supplementary material [file 41598_2019_50447_MOESM1_ESM.pdf]

Temporal processing of past and future autobiographical events in patients with schizophrenia

Hédi Ben Malek, Arnaud D'Argembeau, Mélissa C. Allé, Nicolas Meyer, Jean-Marie Danion & Fabrice Berna

Information S1. Sensitivity analyses.

Sensitivity analyses using pessimistic priors for time orientation (i.e., past vs. future) showed that the group effect for the use of specific events to locate events was no longer meaningful (OR=0.73, CI95%:0.45-1.13, *Pr*(OR>1) = .07). In contrast, conclusions remained unchanged regarding the group effect for the use of factual information (OR=0.57, CI95%:0.35-0.88, *Pr*(OR>1) = .006), and the interaction effect for the use of contextual details (OR=2.66, CI95%:1.28-4.97, *Pr*(OR>1) = .99), for the use of lifetime periods/extended events (OR=0.53, CI95%:0.29-0.90, *Pr*(OR>1) = .01), and for the use of factual information, (OR=1.69, CI95%:0.95-2.88, *Pr*(OR>1) = .95).

Table S2. Mean percentages (and standard deviations) of combinations of temporal location strategies for patients with schizophrenia (n = 30) and controls (n =30).

	Controls n=30		Patients n=30	
	M	*SD*	*M*	*SD*
Lifetime periods/extended events – Specific landmark events	14.76	18.38	18.58	32.83
Lifetime periods/extended events – Conventional time patterns	8.63	13.56	9.21	24.04
Lifetime periods/extended events – Factual knowledge	17.82	20.53	36.88	36.57
Lifetime periods/extended events – Contextual details	31.64	33.65	9.82	13.84
Specific landmark events – Conventional time patterns	3.85	8.16	9.52	25.01
Specific landmark events – Factual knowledge	3.30	6.31	13.03	26.96
Specific landmark events – Contextual details	3.30	6.31	2.63	11.47
Conventional time patterns – Factual knowledge	6.34	11.13	3.95	12.53
Conventional time patterns – Contextual details	1.72	5.61	5.26	13.38
Factual knowledge – Contextual details	11.48	21.09	4.26	10.75

Table S3. Non-informative and pessimistic priors parameters for time orientation (i.e., past vs. future) for the analysis of the frequency of temporal location strategies (based on the results of Ben Malek, Berna & D'Argembeau, 2017).

	Non-informative		Pessimistic	
	Alpha (precision)	Beta (precision)	Alpha (precision)	Beta (precision)
Lifetime periods/extended events	0 (0.01)	0 (0.01)	-0.064 (0.5)	-0.168 (8)
Specific landmark events	0 (0.01)	0 (0.01)	0.006 (0.5)	-0.028 (8)
Conventional time patterns	0 (0.01)	0 (0.01)	-0.009 (0.5)	-0.201 (8)
Factual information	0 (0.01)	0 (0.01)	0.041 (0.5)	0.113 (8)
Contextual details	0 (0.01)	0 (0.01)	-0.155 (0.5)	-1.681 (8)

Note. The regression equation was defined as following: Y = Alpha + Beta * Time orientation

Table S4. Non-informative and pessimistic priors parameters for time orientation (i.e., past vs. future) for the analysis of event characteristics (based on the results of Ben Malek, Berna & D'Argembeau, 2017).

	Non-informative		Pessimistic	
	Alpha (precision)	Beta (precision)	Alpha (precision)	Beta (precision)
Subjective vividness	0 (0.01)	0 (0.01)	0.579 (0.01)	2.166 (0.784)
Affective valence	0 (0.01)	0 (0.01)	-1.654 (0.01)	3.696 (0.512)
Importance for personal goals	0 (0.01)	0 (0.01)	0.360 (0.01)	2.804 (0.574)
Mental time travel	0 (0.01)	0 (0.01)	0.487 (0.01)	2.611 (0.781)
Event rehearsal	0 (0.01)	0 (0.01)	-0.027 (0.01)	4.455 (0.239)
Time rehearsal	0 (0.01)	0 (0.01)	-0.338 (0.01)	2.902 (0.642)
Subjective temporal distance	0 (0.01)	0 (0.01)	-0.283 (0.01)	1.300 (1.921)

Note. The regression equation was defined as following: Y = Alpha + Beta * Time orientation
